# Supplementary material for: Lifestyle and the presence of helminths is associated with gut microbiome composition in Cameroonians
Source: Genome Biol. 2020 May 25;21:122. doi: 10.1186/s13059-020-02020-4 (PMC7249393; doi:10.1186/s13059-020-02020-4)

Fig. S1. ANTS frequency colored by assay type.

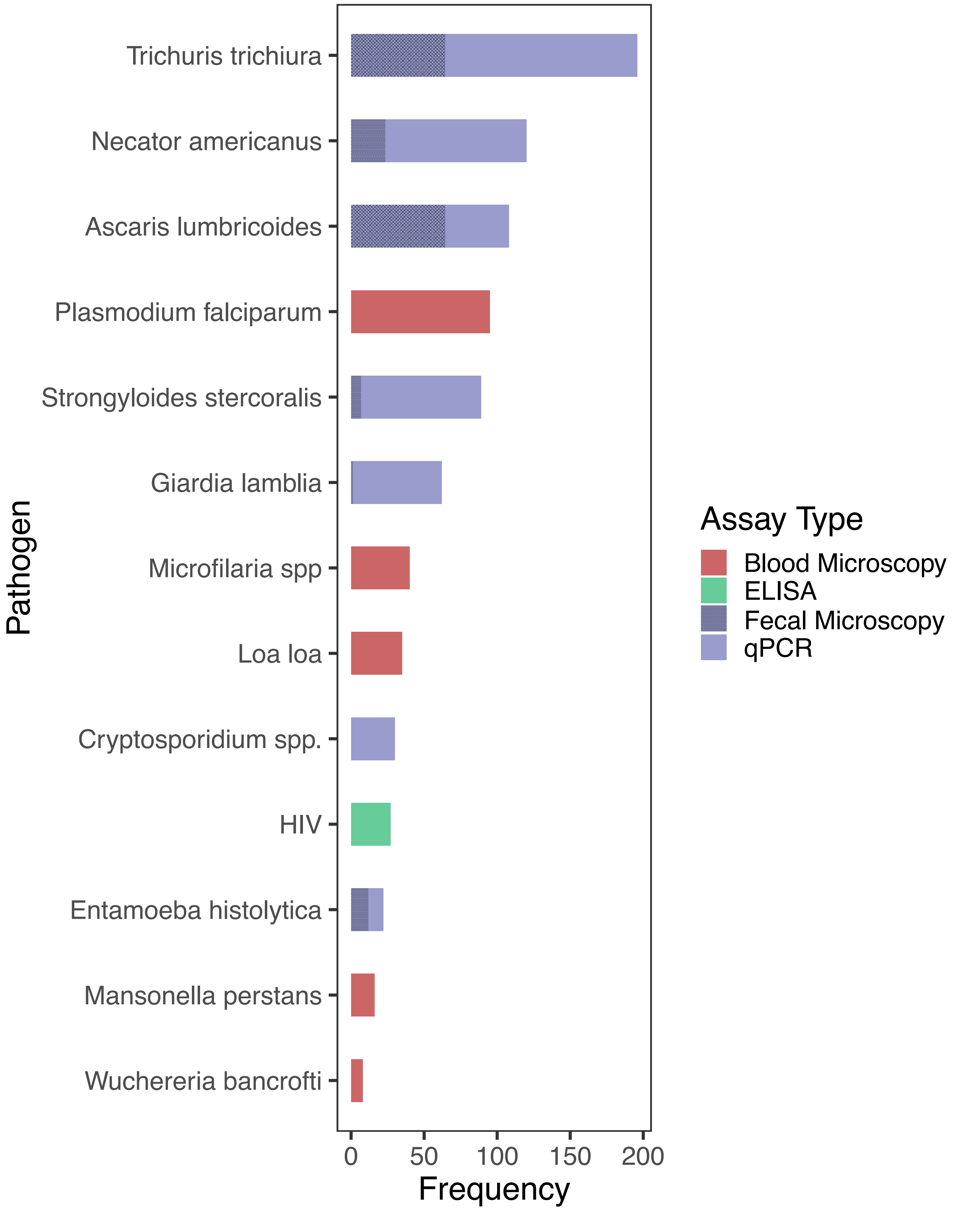

Fig. S2. Positive parasite samples visualized by their qPCR cycle threshold values (Ct), with ANTS parasites on the top row.

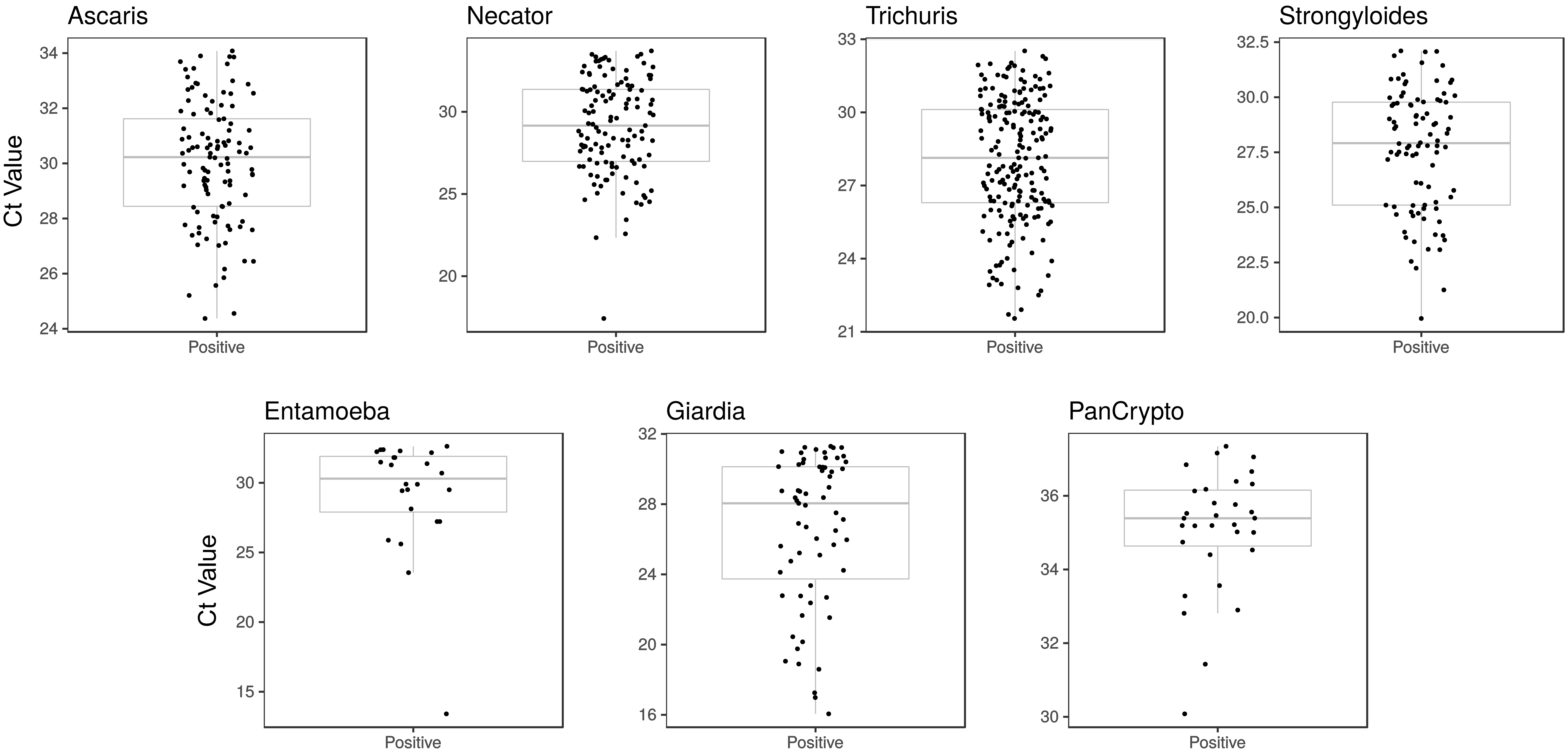

Fig. S3. Correlation plot between metadata variables of interest.

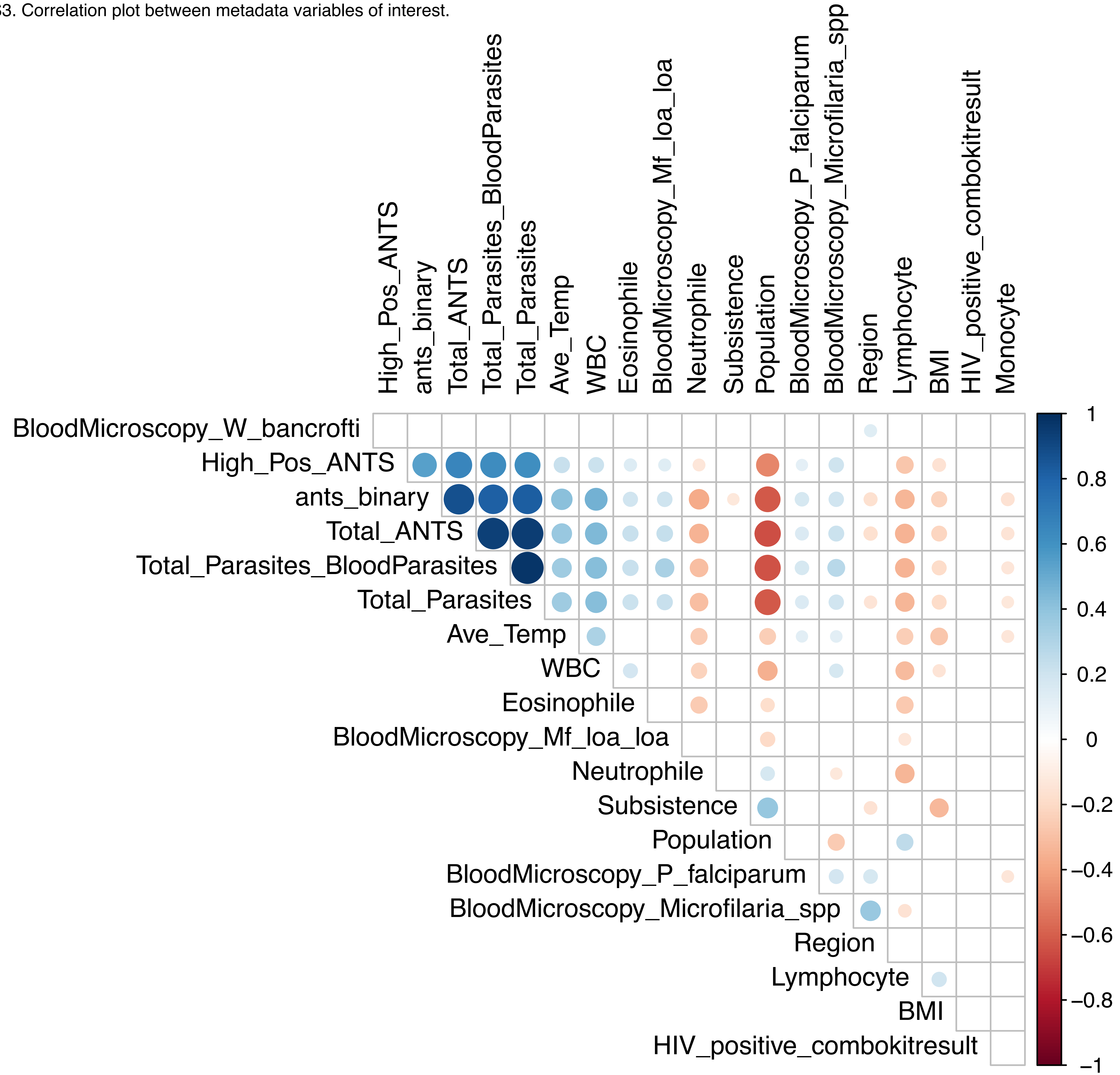

Fig.S4. Bacterial 16S alpha diversity metrics across populations and subsistence groups. A: Faith's Phylogenetic Diversity for U.S. and Cameroon populations. B: Faith's Phylogenetic Diversity for U.S. and Cameroon populations. B: Faith's Phylogenetic Diversity for subsistence groups. C: Richness for U.S. and Cameroon populations. D: Richness for subsistence groups.

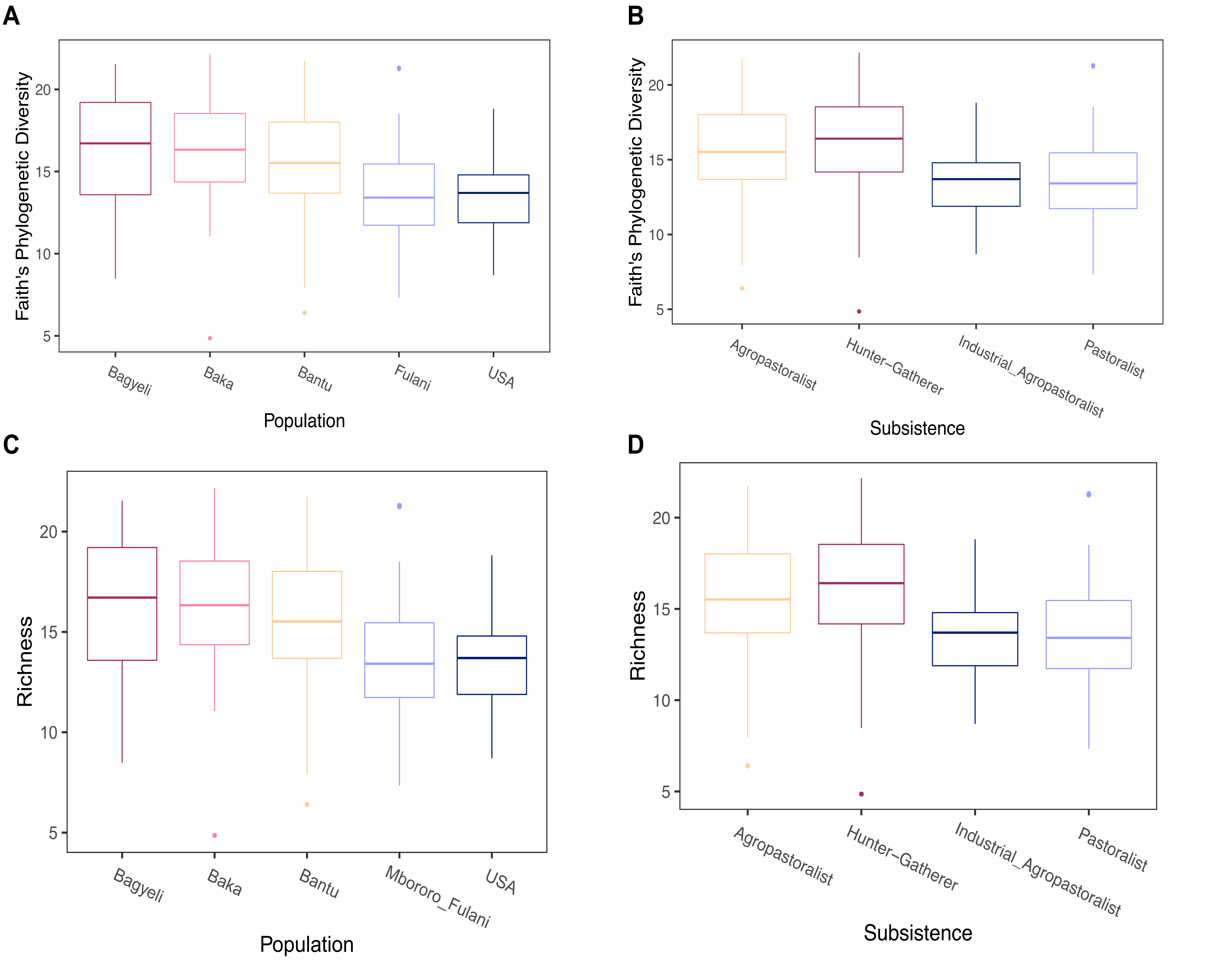

Fig. S5. Most differentially abundant taxa between Bantu regions by bacterial phylum for A: Bantu in Northwest and East regions, B: Bantu in the South and Northwest Regions, and C: Bantu in the South and East Regions.

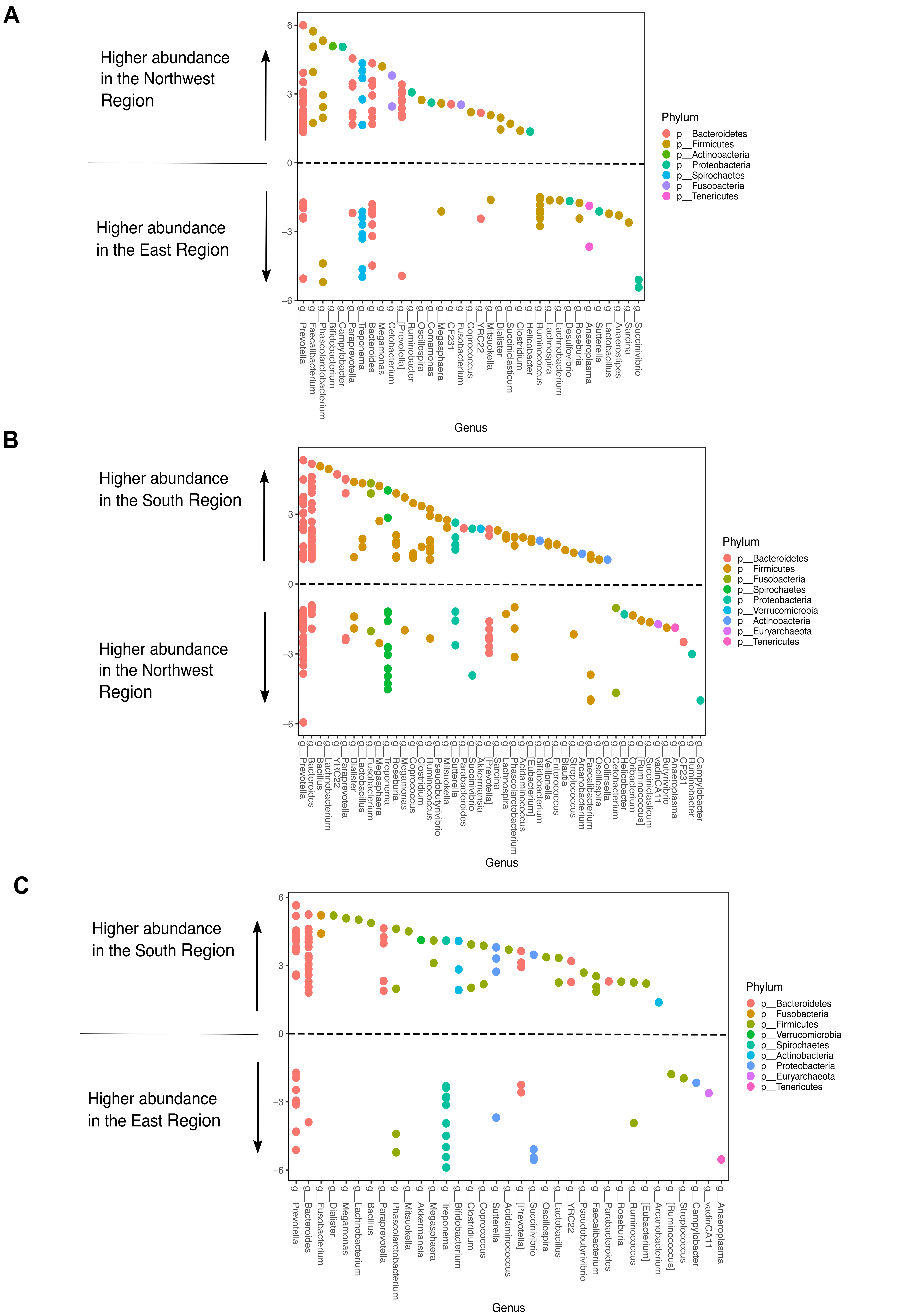

Fig. S6. Top ten most important variables in RFC from 16S rRNA V4 analysis for all Cameroonians with and without ANTS parasites (left), and relative abundances of RFC taxa visualized between positive and negative Cameroonians (right).

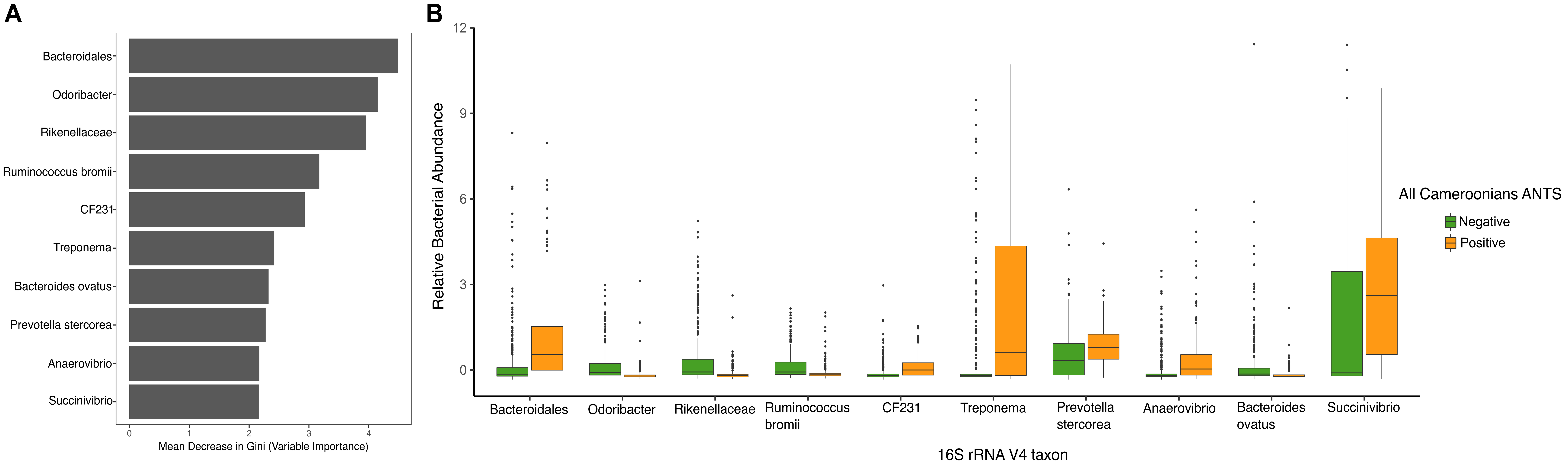

Fig. S7. Shotgun sequencing diversity metrics across Cameroonian populations. A: Simpson Index across all populations. B: Shannon index across all populations.

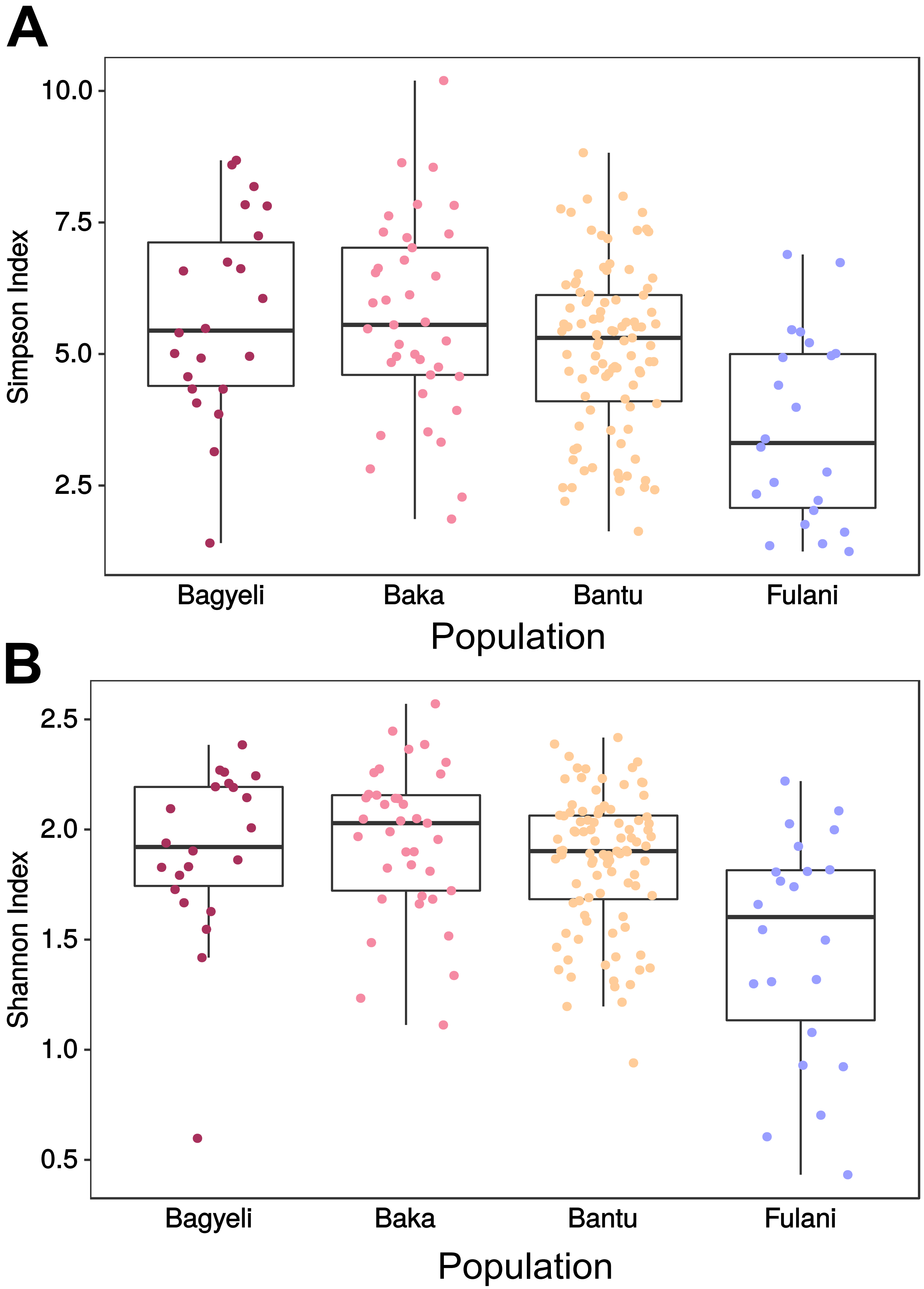

Fig. S8. Comparison of the most abundant 15 taxa proportions in shotgun sequencing and 16S rRNA amplicon sequencing within each Cameroonian population.

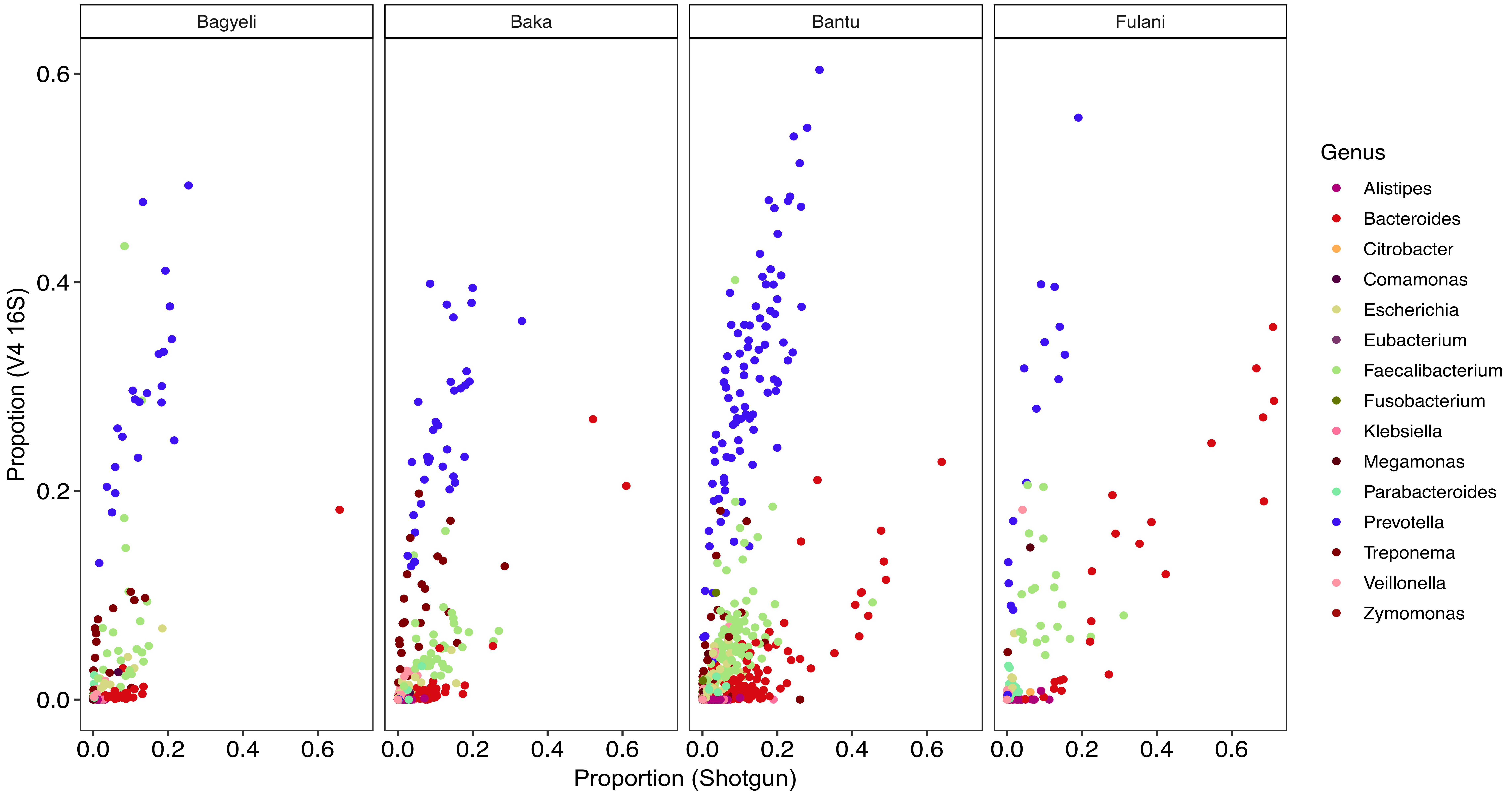

Fig. S9. Relative abundance of reads classified at or more specific than the family-level from Cameroon and HMP samples using a Kraken2 database built using the MAG sequences and taxonomy from Pasolli et al. (2019). Families in the top 10 most-abundant for either the HMP data or the Cameroon data are colored, others are grouped into the "other" category.

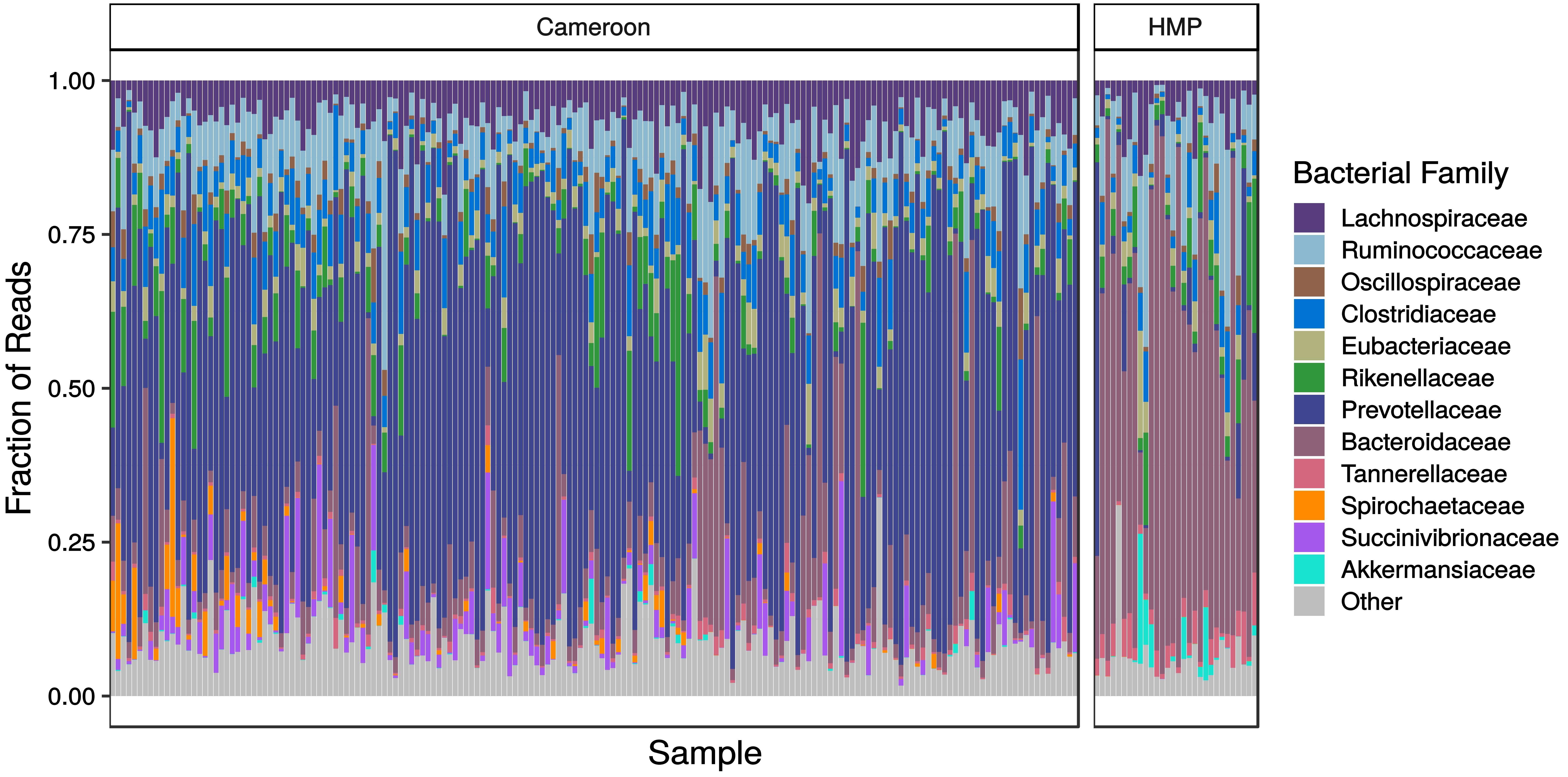

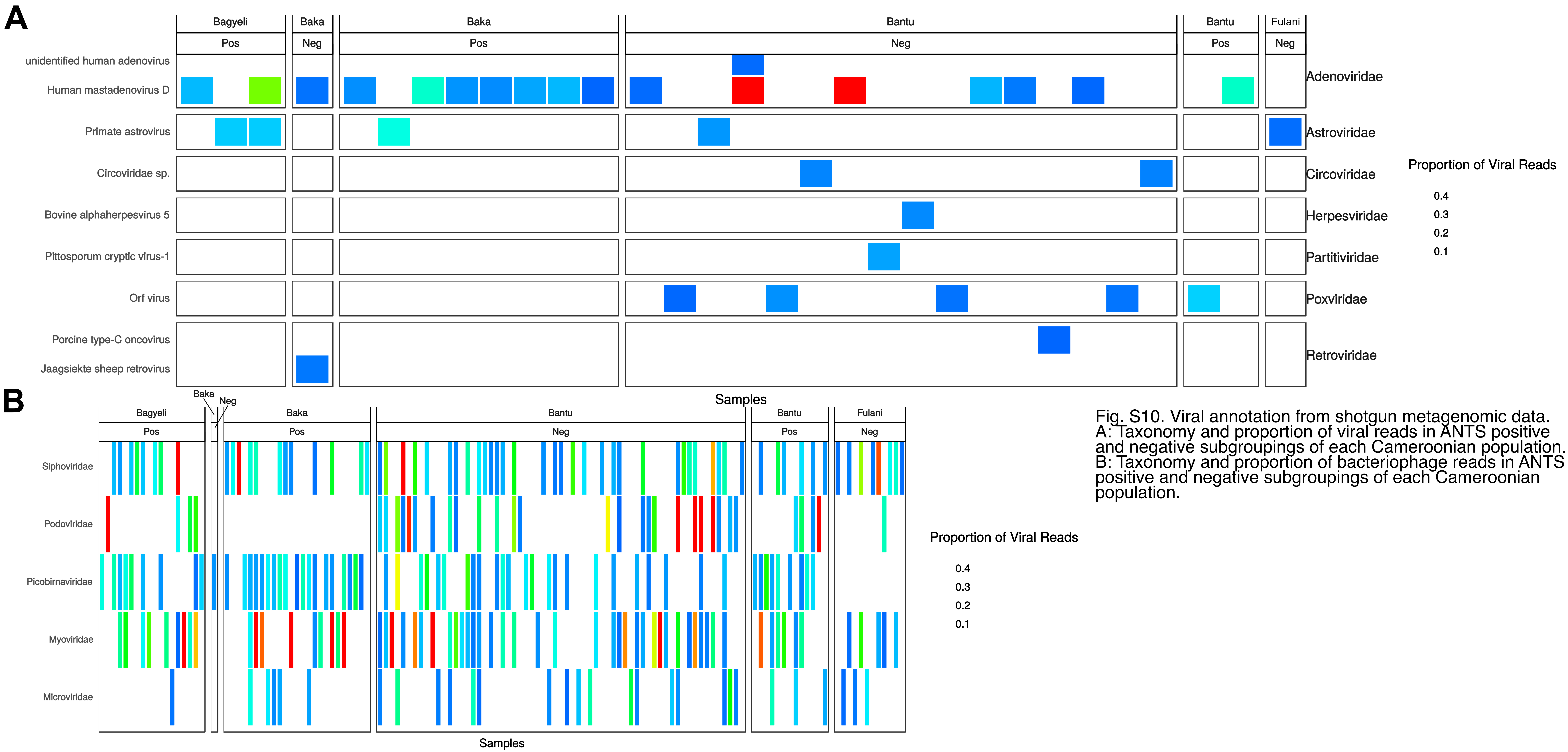


Fig. S10. Viral annotation from shotgun metagenomic data. A: Taxonomy and proportion of viral reads in ANTS positive and negative subgroupings of each Cameroonian population. B: Taxonomy and proportion of bacteriophage reads in ANTS positive and negative subgroupings of each Cameroonian population.

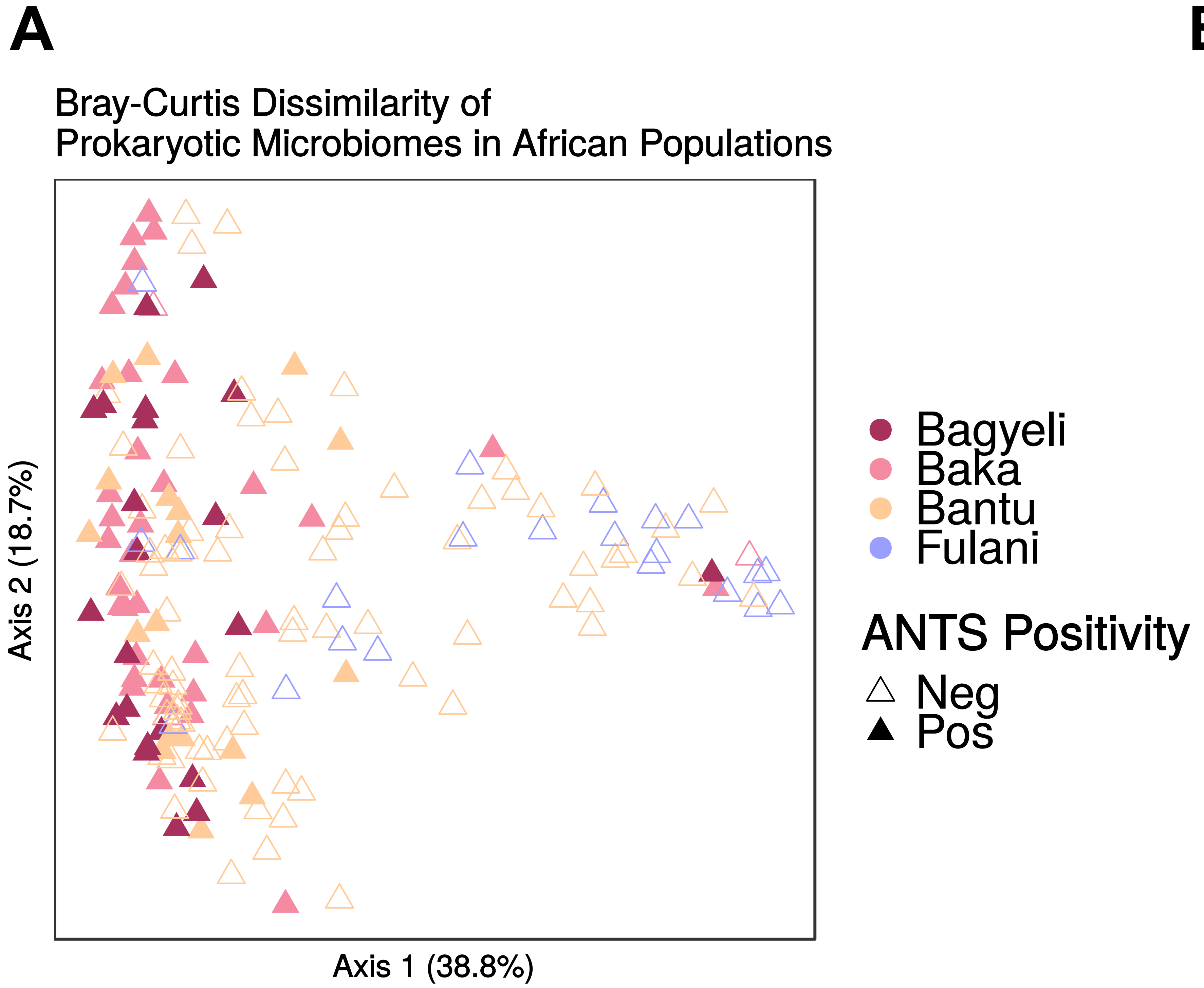


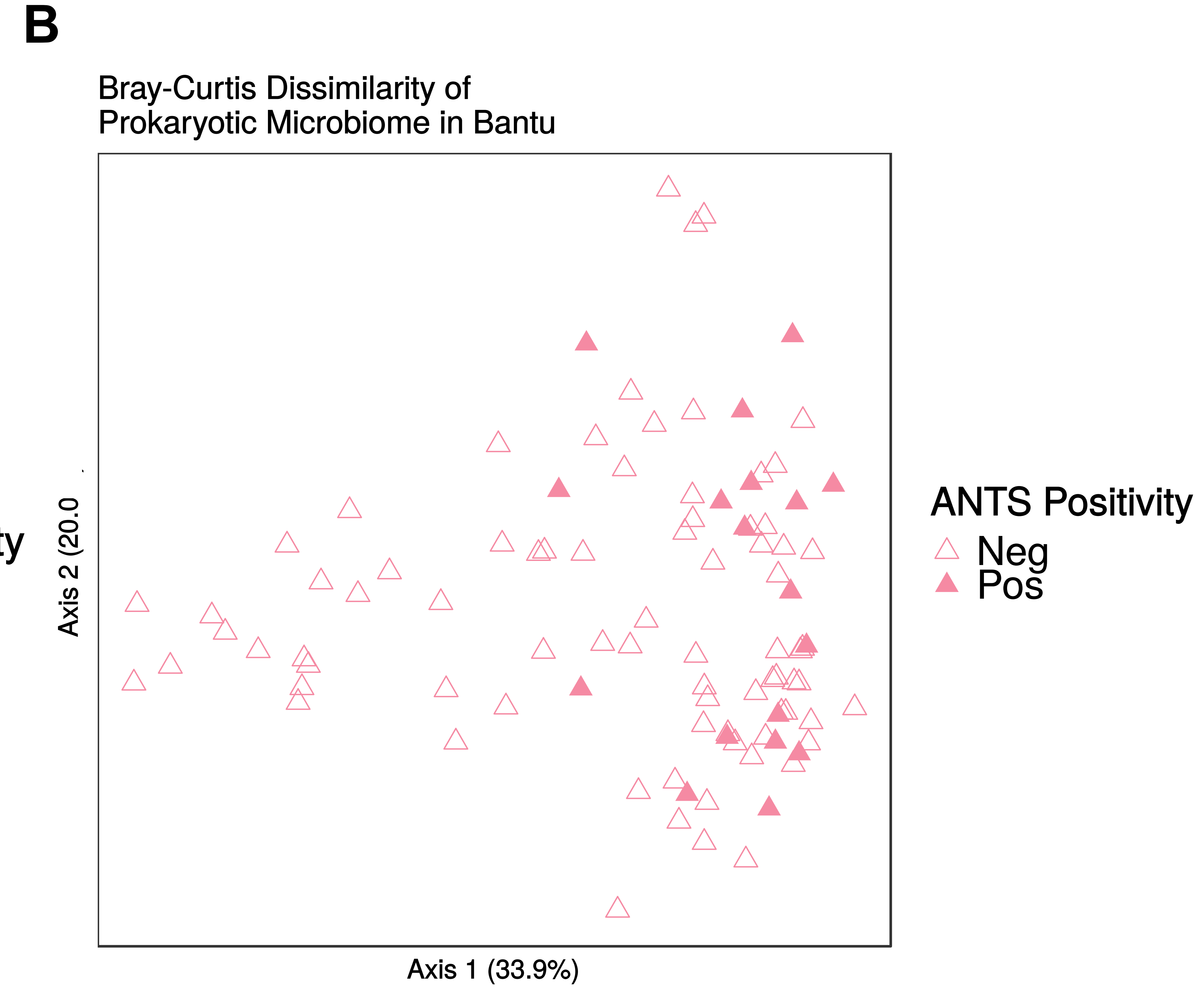


Fig. S11. Bray-Curtis dissimilarity of prokaryotic microbiomes shaded by ANTS positive status in A) African populations and B) in Bantu only.

Fig. S12. Top ten most significant taxa in RFCs on shotgun data for A: Subsistence for all Cameroonians B: ANTS detection (positive/negative) in all Cameroonians C: ANTS detection (positive/negative) in Bantu only.

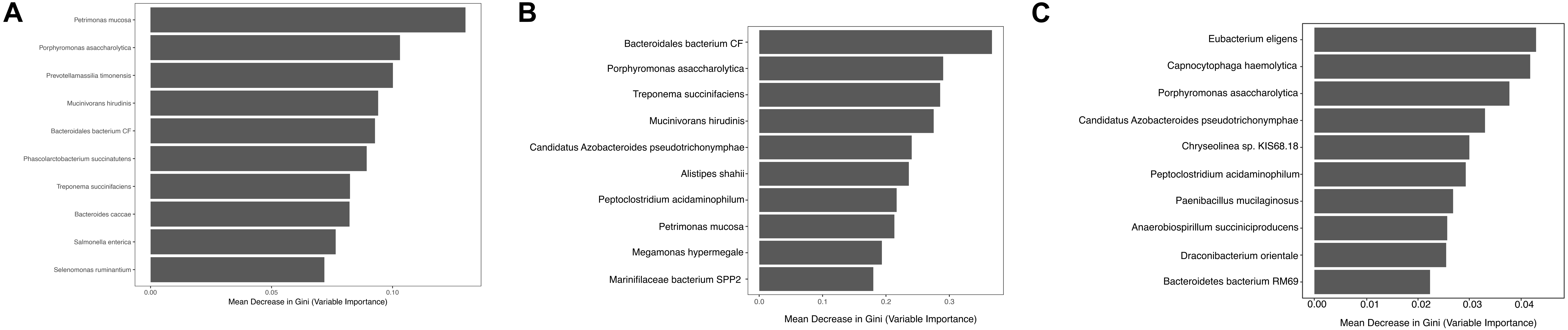

Fig. S13. Correlation plots between cytokine values and pathogen variables of interest and IL-5 cytokine RFC on shotgun data. Negative correlations are displayed in red and positive correlations are shown in blue. Color intensity and the circle size are proportional to correlation coefficients. A: Correlation plot of ANTS detection ("ants_binary") with cytokines. B: Correlation plot of ANTS counts ("Total_ANTS") with cytokines. C: Correlation plot of HIV status ("HIV") with cytokines. D. Correlation plot of *Plasmodium falciparum* detection with cytokines.

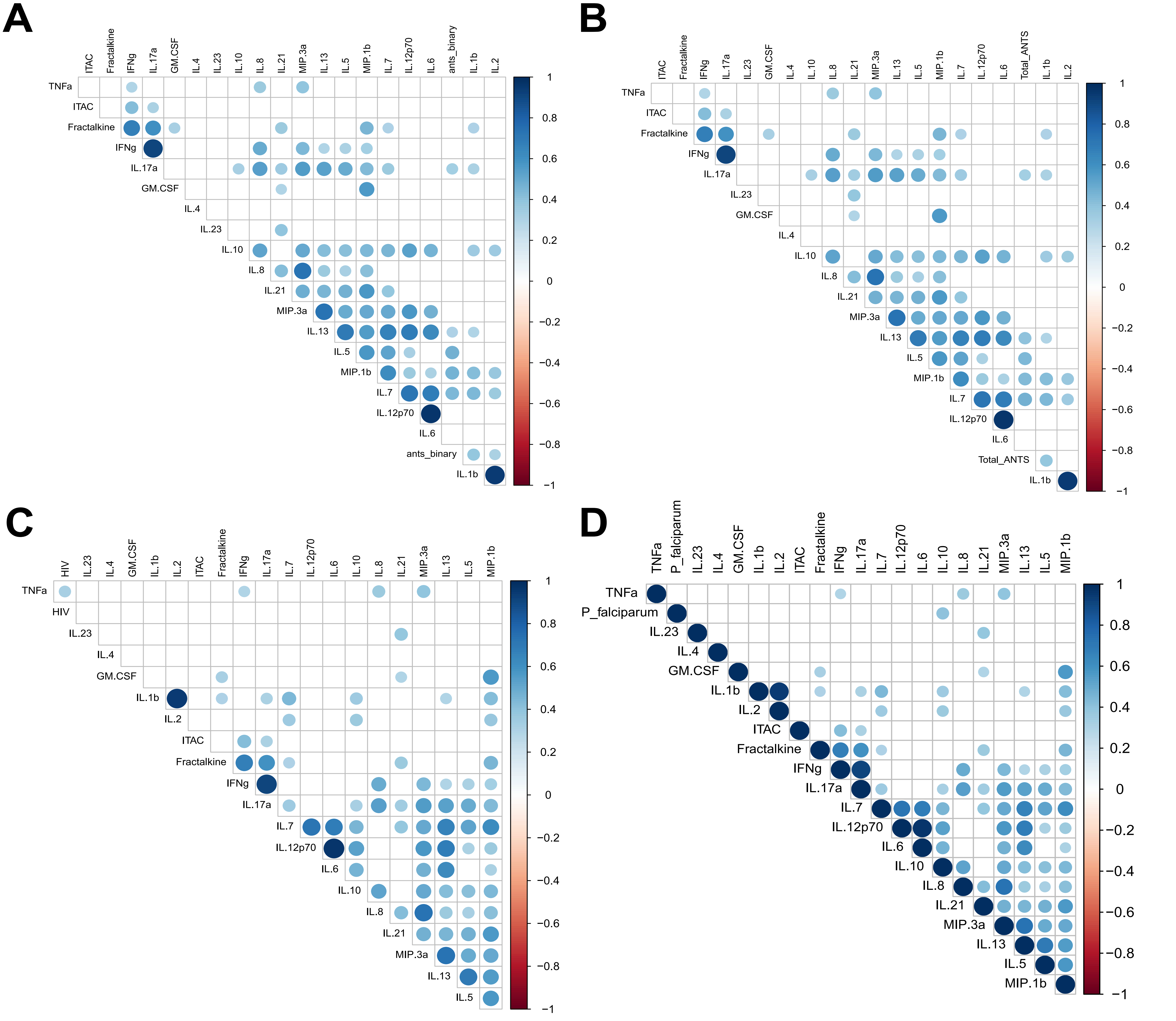

Fig. S14. Top ten most significant taxa in RFCs on shotgun data for IL-5.

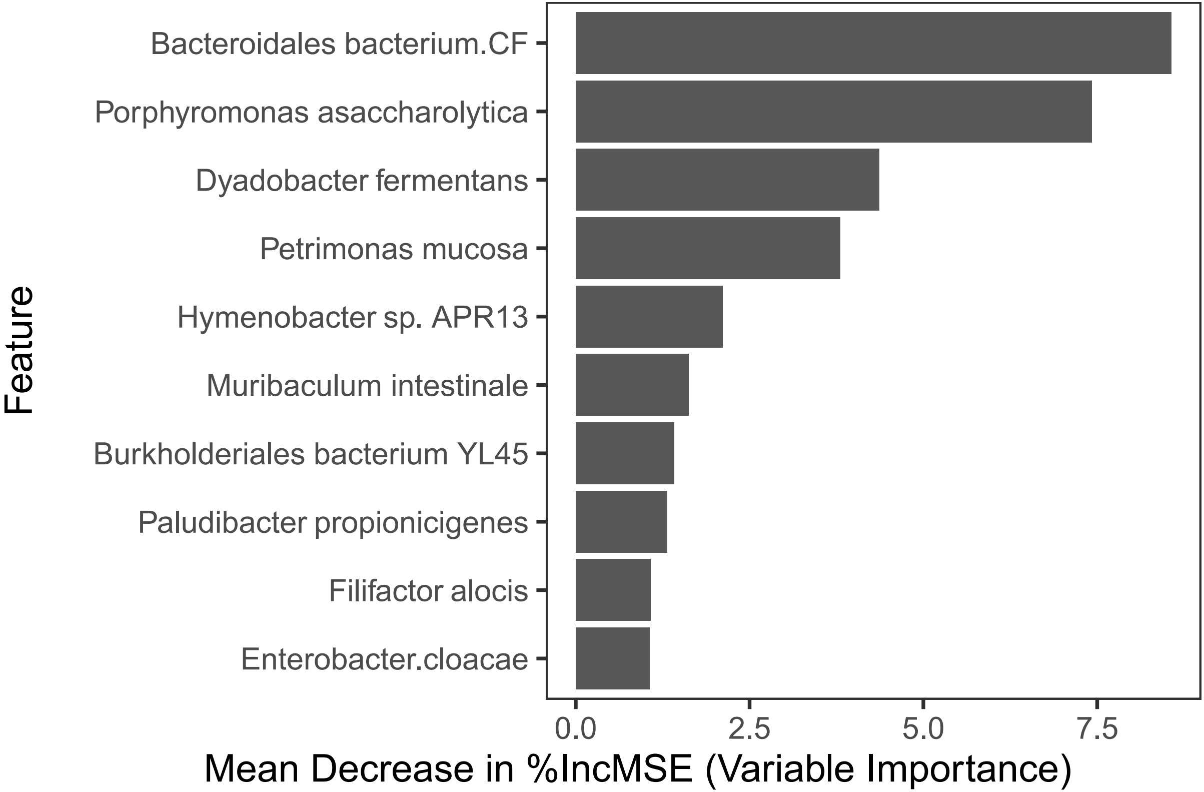

Fig. S15. Lactose metabolism pathways contingent on host lactose digestion phenotype. If humans continue to produce the lactase enzyme after weaning, they are capable of breaking down lactose sugar from dairy. This phenotype is called lactase persistence (LP). In LP individuals (top of the dashed line), the disaccharide lactose will be cleaved by the lactase enzyme into monosaccharides glucose and galactose at brush-border of the small intestine. Glucose and galactose are transferred into absorptive enterocytes, where they then move through the intestinal lumen, across the epithelium and enter the host bloodstream for further metabolism. If humans cease producing the lactase enzyme after weaning, they are not capable of breaking down lactose sugar in the small intestine. This phenotype is called lactase non-persistence (LNP) (bottom of dashed line). In this case, ingested lactose will pass through the small intestine and enter the large intestine. Bacteria possessing the LAC operon are capable of producing the enzyme β-galactosidase. Host colonic bacteria producing β-galactosidases are also capable of catabolizing lactose into glucose and galactose. The fermentation products of this reaction can produce a series of intermediate (e.g., succinate, lactate, and formate) and end-product metabolites (butyrate, propionate, acetate, gases CO2,H2, and CH4).

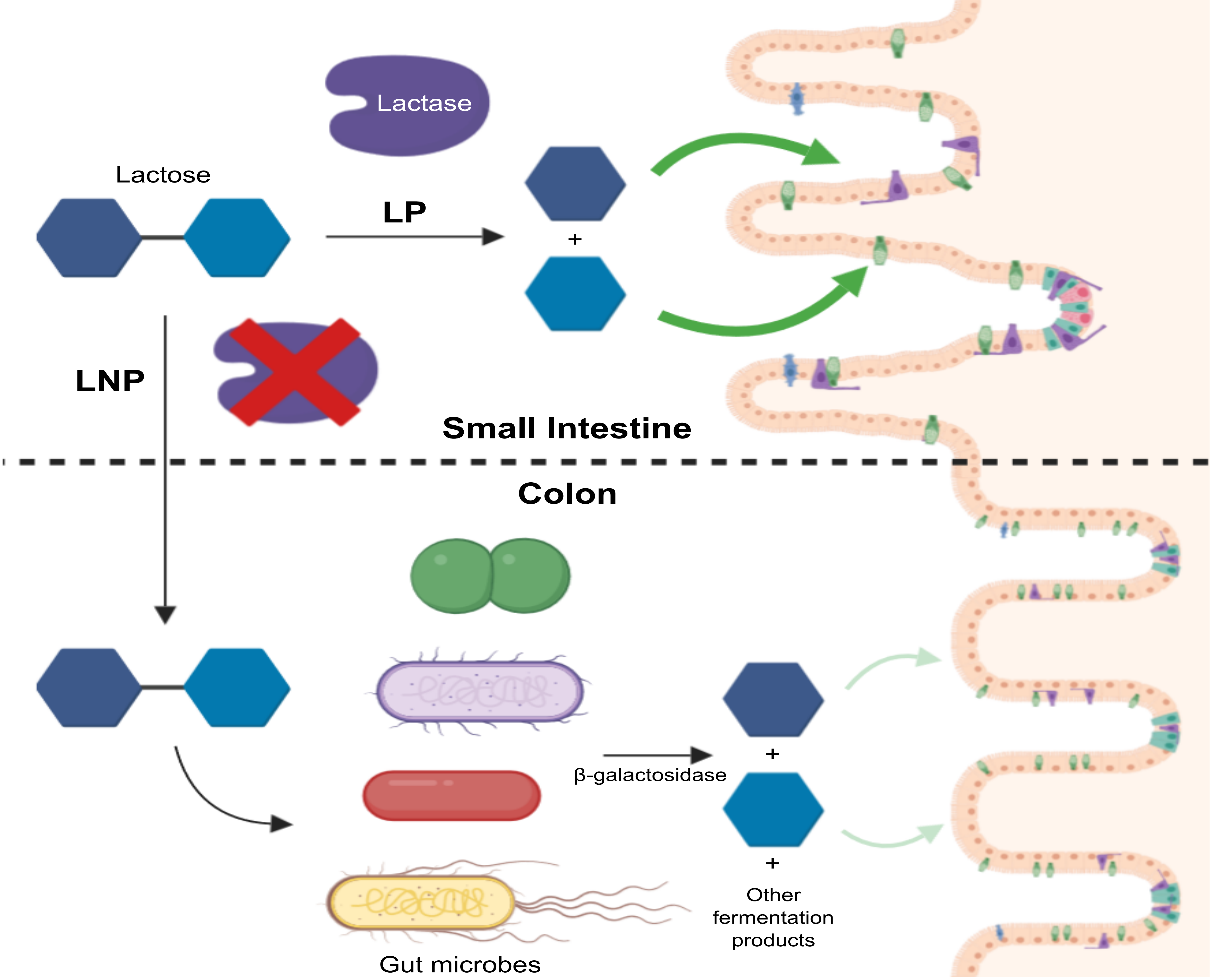

Supplement: Supplementary file 1 — Additional file 1. All supplementary figures. [file 13059_2020_2020_MOESM1_ESM.pdf]
